# Gaze cues facilitate incidental learning in children aged 7–10 years, but arrow cues do not

**DOI:** 10.3758/s13423-025-02657-x

**Published:** 2025-02-14

**Authors:** Mitsuhiko Ishikawa, Ayumi Yoshioka

**Affiliations:** 1 Hitotsubashi Institute for Advanced Study, Hitotsubashi University, 2-1, Naka, Kunitachi, Tokyo, 186-8601 Japan; 2 Research Organization of Science and Technology, Ritsumeikan University, 1-1-1 Noji-Higashi, Kusatsu, Shiga 525-8577 Japan; 3 Section of Brain Function Information, National Institute for Physiological Sciences, 38 Nishigonaka Myodaiji, Okazaki, Aichi 444-8585 Japan

**Keywords:** Gaze cueing, Social learning, Children, Incidental memory

## Abstract

From infancy, humans use gaze cues from others to learn about their surrounding environment. It is known that gaze cues facilitate the cognitive processing of targets in both infants and adults, but what developmental changes occur during childhood? This study investigated the impact of gaze cueing on incidental learning in children aged 7–10 years (n = 80). Participants completed a cueing task where they were presented with gaze or arrow cues (valid or invalid) followed by a target stimulus. Then, participants conducted an incidental memory test including the target stimuli presented in the cueing task and novel stimuli. Results revealed that valid gaze cues significantly enhanced memory performance compared to arrow cues, while gaze cues and arrow cues had similar effects on attention orienting. This effect was consistent across age groups, suggesting that gaze cueing facilitates memory regardless of developmental stage. The findings support the socio-communicative aspect of gaze cues, which may influence cognitive facilitation in joint attentional situations in childhood. Joint attentional situations may be rewarding, influencing the motivation for implicit cognitive processing of objects that are the focus of attention. This study contributes to our understanding of spontaneous social cognition in children and underscores the importance of gaze cues in facilitating memory and learning in social contexts.

**Supplementary Information:**

The online version contains supplementary material available at 10.3758/s13423-025-02657-x.

## Introduction

From early developmental stages, humans use gaze cues to learn about the surrounding environment. Infants, for instance, are known to follow the gaze of adults, which helps them identify significant objects in their surroundings and learn about their environment (Butterworth & Jarrett, 1991). This early-developing skill underscores the importance of gaze in social learning and communication. Research on infants has demonstrated that when an adult's gaze is directed at an object, the infant’s learning about that object is facilitated (Hoehl et al., 2008; Michel et al., 2019; Reid & Striano, 2005; Reid et al., 2004). This suggests that gaze following is a fundamental mechanism through which humans acquire knowledge from others.

As children grow into adults, the role of gaze cues in learning and memory becomes more pronounced. Gaze cues do not merely direct attention; they also seem to enhance the encoding of information. Studies with adults have reported that gaze cues can enhance memory for objects at the cued location (Dodd et al., 2012). Dodd et al. (2012) found that gaze cues facilitate the memory encoding of cued objects, suggesting a deeper cognitive processing of socially cued stimuli. Like gaze, arrow cues can elicit strong orienting of attention detectable both covertly and overtly (Dalmaso et al., 2020a; Tipples, 2008), but the study by Dodd et al. (2012) did not find an effect of enhancing memory for the cued target. Similarly, Kim and Mundy (2012) demonstrated that gaze direction could influence memory performance, indicating that social attention mechanisms are involved in enhancing cognitive processing. In addition to adults, research on older adults has revealed interesting patterns regarding gaze cueing and memory. Although older adults exhibit a reduction in the gaze-cueing effect on attention orienting compared to younger adults, gaze cues still enhance memory encoding (Gregory & Kessler, 2022). This suggests that the fundamental mechanism by which gaze cues enhance memory remains intact throughout the lifespan, though its effects on attention orienting may diminish with age.

In another set of studies investigating gaze cueing effects on the encoding of memory, visual working memory (vWM) was measured. Gregory and Jackson (2017) adapted the classical gaze-cueing paradigm in conjunction with a vWM task to examine how gaze cues affect memory enhancement. Their study involved a central face that gazed either left or right, followed by a memory array of four coloured squares appearing on the cued or uncued side during the encoding interval. Participants were asked to recall whether a probe item had been part of the memory array. The results demonstrated that participants' vWM performance was more accurate in the cued condition compared to the uncued condition, an effect not observed with arrow cues. Gaze cues presented before memory items can shape external representations in the valid cued direction and modulate already-stored internal representations. Nie et al. (2018) used a retro-gaze cue paradigm in a vWM task, in which gaze cues were used as retrospective cues to test if observers could remember a memory array after a non-predictive gaze cue during the maintenance interval. The study found a memory advantage for valid gaze-cued conditions compared to invalid ones. Non-social cues like motion and reverse gaze cues did not similarly affect vWM. These findings emphasize that social cues, such as gaze cues, enhance vWM performance during both encoding and maintenance intervals, playing a crucial role in memory enhancement by allocating attention effectively.

In these vWM tasks using gaze cueing, participants were instructed to remember the target. However, the facilitation effects of gaze cueing, as shown in infant studies (e.g. Reid & Striano, 2005), likely relates to the implicit influence of gaze cueing on information processing, since instructions cannot be given to pre-verbal infants. Thus, although studies with both infants and adults have reported enhanced encoding due to gaze cueing, a distinction remains with regard to whether the cognitive processing involved is implicit or explicit. While implicit cognition, including incidental learning, has been suggested to develop early (around 6 months) and remain relatively stable across the lifespan, research indicates that certain factors such as stimulus type, task design, and measurement methods can influence its effectiveness, especially in children (Amso & Davidow, 2012). Incidental learning refers to the acquisition of knowledge without the intention to learn, often occurring as a byproduct of another activity. This form of learning is particularly relevant in naturalistic settings where children often learn incidentally through interactions with others. Studies have shown that while implicit memory tasks using contextual cueing are performed by a wide age range, including young children and older adults, the performance of around 7-year-olds can be more susceptible to experimental manipulations compared to adults (Dixon et al., 2010; Merrill et al., 2013; Yang & Merrill, 2015). During the 7- to 10-year age range, children experience substantial growth in working memory capacity and strategy use, which has been linked to implicit memory performance (Cowan, 2016; Yang & Merrill, 2018). In this age range, the implicit memory of attentionally cued objects may undergo developmental changes.

Crucially, this age range also encompasses important developments in children's ability to perceive and interpret others' gaze. Research suggests that while younger children can perceive triadic gaze (i.e., averted gaze), the accuracy and sensitivity of this ability continue to improve throughout the elementary school years. Children's gaze-detection thresholds gradually approach adult levels, with significant improvements observed between the ages of 6 and 10 years (Vida & Maurer, 2012). This developmental trajectory is supported by multiple studies examining explicit triadic gaze judgments in children (e.g., Butterworth & Itakura, 2000; Doherty et al., 2009). Additionally, joint attention skills, which involve following and responding to others' gaze, show continued development during this period. Studies have observed linear growth in joint attention abilities in typical children aged 8–10 years (Gulsrud et al., 2014), suggesting that elementary school-aged children become increasingly attuned to the focus of others' attention. These developmental trends raise the possibility that children in the 7- to 10-year age range may exhibit enhanced spontaneous cognitive processing of objects that others are looking at.

On the other hand, while the frequency of joint attention behaviours increases between the ages of 7 and 10 years, there may be no age-related changes in information processing during joint attention. The research that measured brain activity during joint attention in children aged 7–10 years showed that an increase in the superior temporal sulcus (STS) activity during joint attention was observed without age difference (Mosconi et al., 2005). Given that the object of attention during joint attention is expected to be relevant for communicative exchange, information processing of the attended object is enhanced, as reflected by increased activity in the STS (Redcay et al., 2016). A recent study has also shown that the STS is involved in the memory encoding of events during social interactions (Masson et al., 2024). Thus, since there is no change in STS activity during joint attention in children aged 7–10 years, there may be no developmental differences in information processing during joint attention. The extent to which this developing gaze sensitivity and joint attention engagement influences spontaneous cognitive processing of the gaze target remains to be fully explored.

This age range is particularly significant in Japan, where elementary school begins at age 7 years, marking a critical period of cognitive and social development. By focusing on this age group, which aligns with the early years of elementary education in Japan, we can investigate whether the developmental progression in gaze perception is mirrored by changes in how gaze cues affect incidental learning. This research could shed light on the complex relationship between social cognitive development and memory processes during a pivotal stage of childhood.

In the current study, we aimed to investigate how gaze cueing affects incidental learning in children aged 7–10 years. By comparing the effects of gaze and arrow cues on incidental learning, we sought to understand the specific contribution of social cues to memory enhancement in children. To examine developmental changes, we divided our participants into two age groups: younger (7–8 years) and older (9–10 years) groups. This allowed us to explore whether the effect of gaze cueing on incidental learning varies with age. We hypothesised that gaze cues would enhance incidental learning more effectively than arrow cues in both age groups. Furthermore, as the increase in joint attention behaviours with development has been reported (Gulsrud et al., 2014), one might expect the effect of gaze cueing on implicit memory enhancement to be stronger in the older group compared to the younger group. On the other hand, given that STS activity during joint attention does not vary within this age range (Mosconi et al., 2005), it is also possible that the effect of gaze cueing on implicit memory may not differ between the age groups.

## Materials and methods

### Participants

The final sample consisted of eighty 7- to 10-year-old Japanese children (41 female, 39 male; mean age = 8.51 years, SD = 1.11 years, range = 84–131 months). Children were recruited with their caregivers at the Japan National Museum of Emerging Science and Innovation (Miraikan), in Tokyo, Japan. Each participant participated in the experiment in an isolated, quiet experimental booth. To examine developmental changes, 40 children aged 7–8 years were designated as the younger group, and 40 children aged 9–10 years were designated as the older group.

The post hoc power analysis was performed using the ANOVA repeated-measures, within-between interaction option in G*Power (Erdfelder et al., 1996), which is appropriate for detecting interactions in mixed designs. The result of the power analysis using a moderate effect size (f = 0.2), alpha level 0.05, and power level 0.95, indicated that a total sample size of 56 participants would be required to achieve sufficient power for detecting the interaction effect (stimuli [gaze, arrow] x cueing validity [valid, invalid] x group [younger, older]), with each between-subject group consisting of 28 participants. This sample size ensures that our study is adequately powered to detect meaningful interaction effects, minimizing the risk of Type II errors.

Preliminary analyses of reaction times in the cueing task and memory performance were conducted with data from 60 participants, consisting of 30 participants per group. To ensure transparency and confirm that this is not a case of p-hacking, the analysis results for all 60 participants are provided in the Online Supplementary Material. Furthermore, since the effect of gaze cueing on implicit memory in children has not been examined, we collected more data than the estimated sample size to ensure robustness in this study.

An additional six children were excluded from the analysis because they either did not complete the experiment or did not follow the instructions.

### Materials

The experiment was performed using PsychoPy 3.2.3 (Peirce, 2007). Stimuli were presented on a laptop (13 in.). Before the trials, a fixation cross was presented in the centre of the screen. For the facial stimuli, we used images of two different Asian adult female faces. Racial group membership has been shown to influence attention orienting via gaze cueing (see review by Dalmaso et al., 2020b). To minimise the influence of potential confounding variables, faces of the same ethnicity as the participants were used as stimuli. In the previous study, these female faces were perceived to be equally attractive (Ishikawa et al., 2021). Gaze cues were preceded by the same face with its eyes directed to the viewer. For the cueing stimuli, the faces were presented with eyes gazing either at the left or the right side (gaze condition). The arrow-cueing stimulus was preceded by a black horizontal line (arrow condition). The arrow cues were black arrows that pointed to the left or right. The target stimulus, presented after the cueing stimuli, was a fractal figure. The fractal figures were randomly generated in terms of colour and shape.　We created 80 fractals in total. The fractal figures were randomly assigned to each condition for each participant.

### Procedure

After obtaining informed consent, the children began with the cueing task.

At the beginning of the task, the children were instructed to respond as quickly and accurately as possible by pressing a key at the location where the colourful fractal figure, the target, was presented. There were two factors for each child, two levels of stimuli (gaze and arrow) and two levels of cueing validity (valid and invalid). Each participant completed 40 trials (ten trials for each condition) in addition to four practice trials. The target stimuli were selected from 80 different fractal figures, with 40 randomly assigned to each condition. The fractal figures assigned to each condition were presented as targets only once during the cueing task. The total number of trials was determined based on a preliminary study to ensure that children within this age range could complete the task. The presentation order of stimuli was pseudo-random, with the constraint of no more than three consecutive trials of the same condition.

**Fig. 1:**
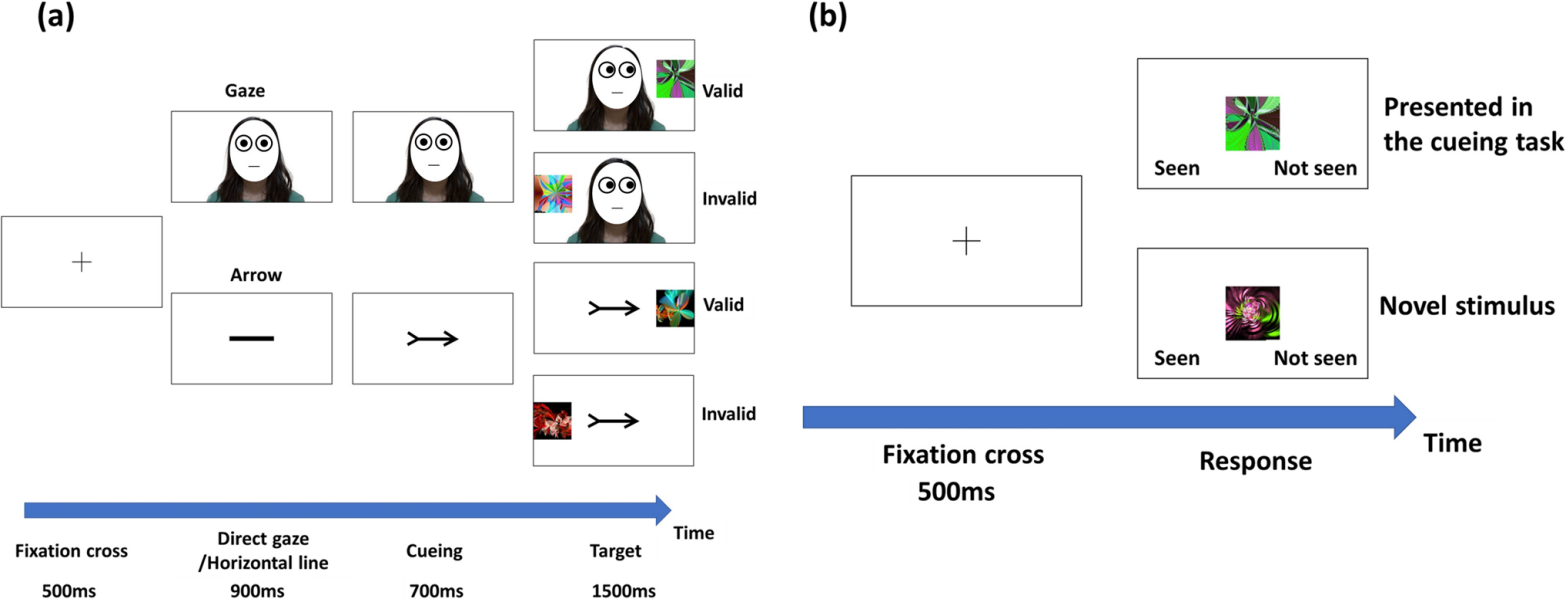
Schematic examples of experimental tasks. (**a**) An example of a trial sequence in the cueing task. (**b**) An example of a trial in the memory test

Figure 1a shows a schematic example of a trial. After the fixation cross was presented for 500 ms, a face with direct gaze or a black horizontal line was presented for 900 ms. Then, a cueing stimulus (eye gaze or arrow) pointing either to the right or to the left, was presented for 700 ms. In a previous study involving children aged 8–13 years, it was shown that there was no difference in the gaze-cueing effect between a stimulus-onset asynchrony (SOA) of 200 ms and a SOA of 700 ms (Goldberg et al., 2008). Therefore, in this study, we set the SOA only to 700 ms.

It was emphasised during the instructions that the direction of the cueing stimuli was not relevant to the target position. The target was presented immediately after the offset of the cueing stimuli. Participants were required to press, as quickly as possible, the ‘Z’ key when the target appeared on the left and the ‘M’ key when the target appeared on the right. The target was displayed for 1,500 ms regardless of the participant’s response. To ensure that the exposure time to each target stimulus was equal for the memory test, the presentation time was kept constant.

After the cueing task, a memory test was conducted immediately (Fig. 1b). After a fixation cross (500 ms), participants were asked to determine whether the fractal figure presented in the centre of the screen had been shown during the cueing task. In the cueing task, participants were not asked to remember the targets, nor were they informed about the memory test following the cueing task. Therefore, this task measured incidental learning. Participants were required to press 'Z' if the fractal figure had been presented during the cueing task and 'M' if it had not.

### Data analysis

For the analysis of the cueing task, stimuli (gaze and arrow) and cueing validity (valid and invalid) were used as independent variables. We computed the number of errors and average response times (RTs) for each condition. Trials in which the participants did not respond correctly to the target location, as well as trials where the RT deviated by more than 3 *SD*s from the participant's mean, were excluded from the RT analysis. A total of 111 trials, accounting for 3.47% of all trials (younger group: 66 trials; older group: 45 trials), were excluded from the calculation of the mean RT.

For the analysis of the memory test, the number of hits (i.e., the number of correctly recognised figures) for each condition was counted for each participant. Also, if all stimuli were judged as presented, the number of hits would be high, so we also calculated and analysed the number of false positives for each condition. Additionally, for a more refined analysis of memory performance, *d*' was calculated by subtracting the z-transformed false positive rate from the z-transformed hit rate for each condition.

## Results

### Performance in the cueing task

The average accuracy rate was quite high for each group (younger group: 96.63%; older group: 97.28%). The children included in the analysis could perform the cueing task as instructed.

**Fig. 2:**
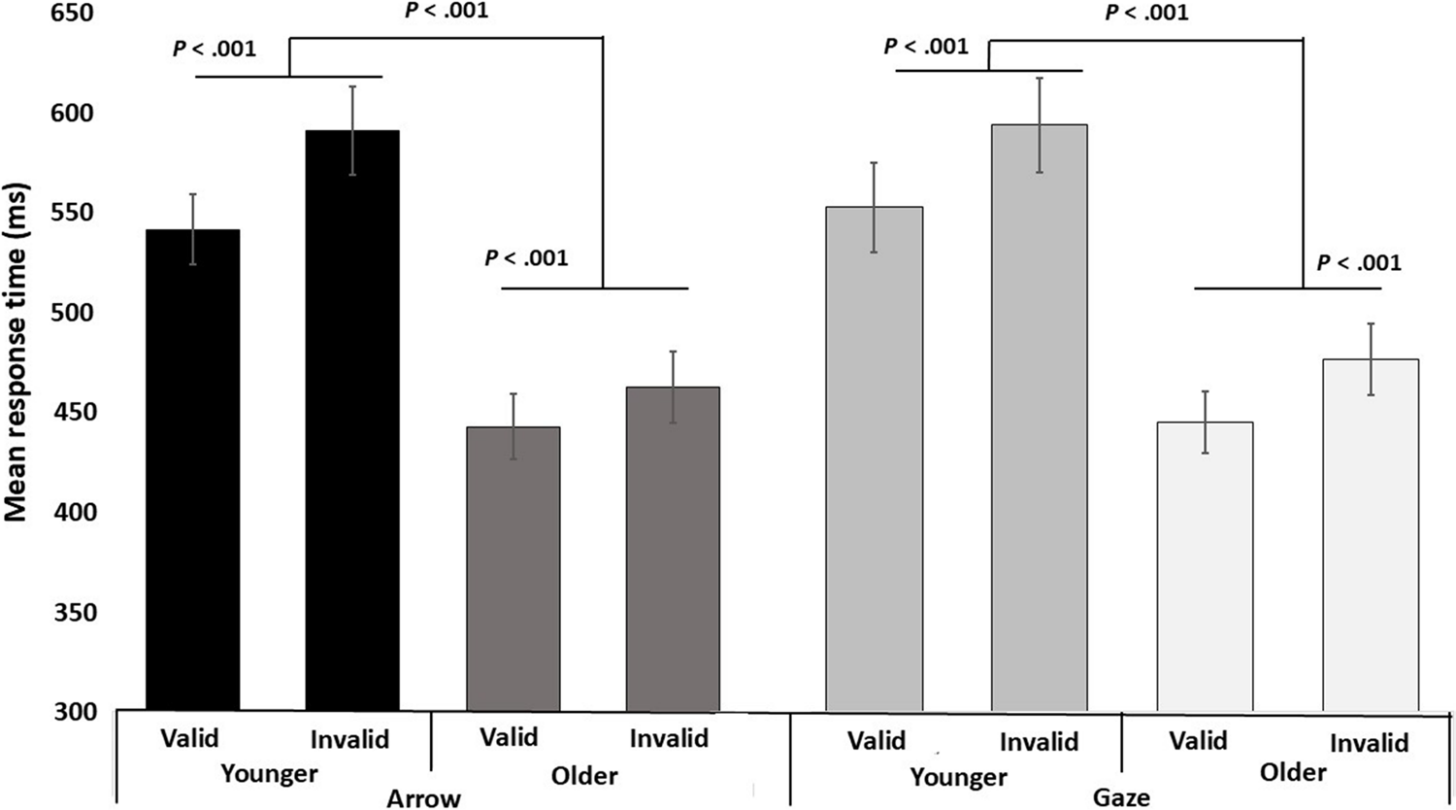
Mean response time for each condition in the cueing task. Error trials and outliers were excluded from the average calculations

A three-way ANOVA (stimuli x cueing validity x group) was carried out on the RTs (Fig. 2). The main effect of cueing validity was found to be significant (*F*(1, 78) = 29.205, *p* < 0.001, *ηp*^*2*^ = 0.272). The children showed shorter RTs in the valid condition compared to the invalid condition (Fig. 2). Also, the main effect of group was significant, showing faster RTs in the older group compared to the younger group (*F*(1, 78) = 20.047, *p* < 0.001, *ηp*^*2*^ = 0.205). The main effect of stimuli was not significant (*F*(1, 78) = 0.091, *p* = 0.763, *ηp*^*2*^ = 0.001). There were no significant interactions (stimuli x group: *F*(1, 78) = 0.091, *p* = 0.764, *ηp*^*2*^ = 0.001; cueing validity x group: *F*(1, 78) = 2.35, *p* = 0.129, *ηp*^*2*^ = 0.001; stimuli x cueing validity: *F*(1, 78) = 0.004, *p* = 0.948, *ηp*^*2*^ < 0.001; stimuli x cueing validity x group: *F*(1, 78) = 1.192, *p* = 0.278, *ηp*^*2*^ = 0.015).

All effects that were significant in this analysis were also observed in the preliminary analysis conducted with 60 participants.

### Memory accuracy

**Fig. 3:**
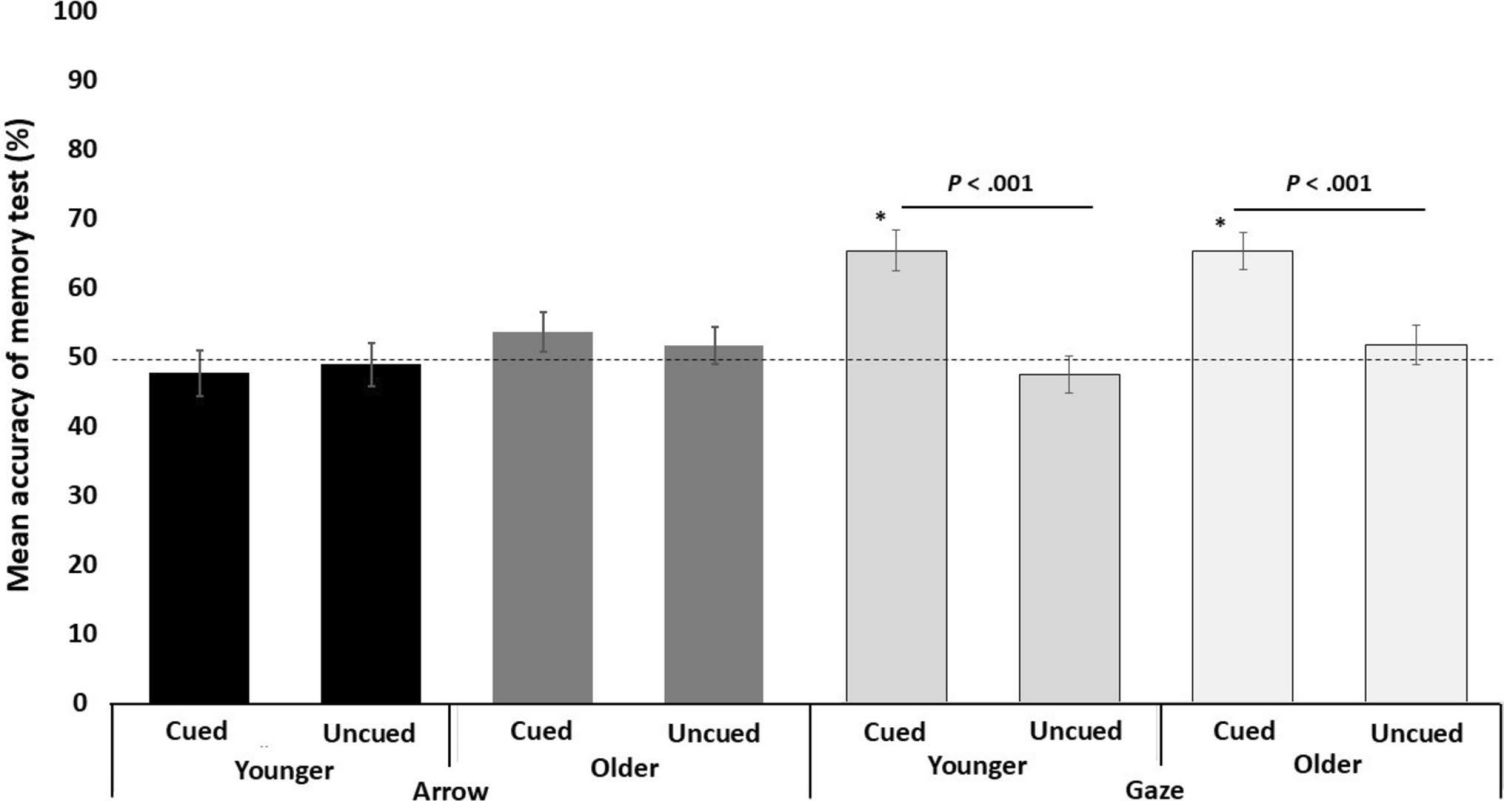
Mean accuracy for each condition in the memory test

A three-way ANOVA (stimuli x cueing validity x group) was carried out on the number of hits (Fig. 3). The main effect of stimuli (*F*(1, 78) = 13.314, *p* < 0.001, *ηp*^*2*^ = 0.146) and the main effect of cueing validity (*F*(1, 78) = 25.809, *p* < 0.001, *ηp*^*2*^ = 0.249) were significant. The main effect of group was not significant (*F*(1, 78) = 1.072, *p* = 0.304, *ηp*^*2*^ = 0.014).

A significant interaction effect between stimuli and cueing validity was found (*F*(1, 78) = 44.97, *p* < 0.001, *ηp*^*2*^ = 0.366). Bonferroni-adjusted post hoc t-tests showed that there were more hits in the gaze-valid condition than in the gaze-invalid condition (*p* < 0.001, *ηp*^*2*^ = 0.467). In the arrow condition, there was no significant difference between the valid and invalid conditions (*p* = 0.851, *ηp*^*2*^ = 0.005). There were no other significant interactions (stimuli x group: *F*(1, 78) = 0.415, *p* = 0.522, *ηp*^*2*^ = 0.005; cueing validity x group: *F*(1, 78) = 0.025, *p* = 0.874, *ηp*^*2*^ < 0.001; stimuli x cueing validity x group: *F*(1, 78) = 2.719, *p* = 0.103, *ηp*^*2*^ = 0.034).

All effects that were significant in this analysis were also observed in the preliminary analysis conducted with 60 participants.

We also conducted t-tests comparing the number of hits in each condition with the chance level. In the gaze-valid condition, both age groups showed higher memory performance than the chance level (younger group: *t(*78) = 5.11, *p* < 0.001, *d* = 0.808; older group: *t*(78) = 5.64, *p* < 0.001, *d* = 0.892). No differences from the chance level were observed in the other conditions (Table 1).

**Table 1:** Statistical values for the comparison of memory performance and chance level in each condition

	Arrow		Gaze	
	Valid	Invalid	Valid	Invalid
Younger	47.75% (*p* = 0.506, *d* = 0.106)	49% (*p* = 0.755, *d* = 0.049)	64.75% (*p* < 0.001, *d* = 0.808)	47% (*p* = 0.266, *d* = 0.178)
Older	53.75% (*p* = 0.190, *d* = 0.210)	51.75% (*p* = 0.524, *d* = 0.102)	65% (*p* < 0.001, *d* = 0.892)	51.25% (*p* = 0.655, *d* = 0.071)

The number of false positives (i.e., when novel fractal figures were recognised as having been seen in the cueing task) might change with development. We compared the number of false positives between age groups using an independent t-test. There was no significant difference between age groups (*t*(78) = 0.239, *p* = 0.811, *d* = 0.038, younger mean = 14.27, SD = 5.65; older mean = 13.97, SD = 5.54).

### Comparison of d'

The *d*' values were compared using a three-way ANOVA (stimuli x cueing validity x group). The main effect of stimuli (*F*(1, 78) = 13.48, *p* < 0.001, *ηp*^*2*^ = 0.147) and the main effect of cueing validity (*F*(1, 78) = 14.18, *p* < 0.001, *ηp*^*2*^ = 0.154) were significant. The main effect of group was not significant (*F*(1, 78) = 1.7895, *p* = 0.1849, *ηp*^*2*^ = 0.022).

A significant interaction effect between stimuli and cueing validity was found (*F*(1, 78) = 29.01, *p* < 0.001, *ηp*^*2*^ = 0.271). Bonferroni-adjusted post hoc t-tests showed that *d*' was significantly higher in the gaze valid condition compared to the gaze invalid condition (*p* < 0.001, ηp^2^ = 0.37). In the arrow condition, there was no significant difference between the valid and invalid conditions (*p* = 0.989, *ηp*^*2*^ = 0). There were no other significant interactions (stimuli x group: *F*(1, 78) = 0.415, *p* = 0.522, *ηp*^*2*^ = 0.006; cueing validity x group: *F*(1, 78) = 0.136, *p* = 0.713, *ηp*^*2*^ = 0.002; stimuli x cueing validity x group: *F*(1, 78) = 0.697, *p* = 0.406, *ηp*^*2*^ = 0.009).

## Discussion

The current study aimed to investigate how gaze-cueing affects incidental learning of the cued targets in children. The results showed that valid gaze cueing at the target location enhanced performance on the memory test conducted after the cueing task. This cueing effect was not observed with arrow cueing. Additionally, there was no difference in the effect of gaze cueing on incidental learning between the younger group and the older group, indicating that the specific effect of gaze cueing was present regardless of the general development of memory abilities. Thus, the enhancement of memory by gaze cues appears to be a phenomenon observed in children aged 7–10 years, independent of the development of social attention and implicit memory.

Why did gaze cueing enhance implicit memory related to the target? It is possible that the communicative aspect of gaze cues had an influence on cognitive facilitation. Bayliss et al. (2006) reported that adult participants showed an increased preference for objects gazed at, an effect not observed with arrow cues. The influence of gaze cueing on target preference should be discussed with caution, as it was not observed in more recent studies with larger sample sizes (Tipples & Pecchinenda, 2018). Although the discussion regarding gaze-liking effects is ongoing, it is suggested that gaze cues act as signals of interest and desire, thereby influencing the perceived value of the cued targets. Such effects of gaze cues on preference are evident from infancy (Ishikawa & Itakura, 2018; Ishikawa et al., 2019). Joint attention elicited by gaze cues may facilitate cognitive processing by allowing individuals to share interest with others. It has been argued that sharing interest with others is inherently rewarding for humans (Tomasello et al., 2005). Furthermore, EEG studies in infants have shown that when gaze cues predict scenarios resembling joint attention, there is an increase in the stimulus preceding negativity (SPN), an ERP component reflecting reward anticipation (Ishikawa & Itakura, 2022). When gaze cues are directed at a target, the communicative signal of the gaze may enhance implicit social motivation, thereby facilitating cognitive processing related to the target.

Ishikawa and Senju (2023) proposed that two types of value influence the motivation to engage in joint attentional situations. The first is informational value, derived from the information gained by following another's gaze. From infancy, others' gaze is used for learning about the environment, making the information obtained by following gaze cues rewarding. The second is social value, which explains the reward inherent in directing attention to the same object as another person. According to the Action Value Calculator Model (Ishikawa & Senju, 2023), these two values can coexist and are not mutually exclusive; the total value determines engagement in social interactions like joint attention. This value-based social motivation operates as an implicit process and may lead to the enhanced cognitive processing observed in joint attention scenarios in this study. Investigating the value and motivation associated with joint attention in atypical populations may deepen our understanding of these mechanisms. For example, in deaf children, the gaze-cueing effect is stronger than in typical children (Pavani et al., 2019). This could be interpreted as gaze direction being more crucial for communication and environmental learning in deaf children. Their everyday experiences might have heightened the value of following another's gaze direction, thereby intensifying its impact on attention orienting. Conversely, it has been argued that social motivation is reduced in individuals with autism, known as the social motivation theory (Chevallier et al., 2012). In these populations, differing motivations for joint attention may lead to different impacts of gaze cueing on implicit memory. Further investigation is needed to determine whether the cognitive processing facilitation effects of gaze cueing vary according to differences in the value of joint attention.

The cueing effect on incidental learning was observed only with the gaze cues, while no difference was found between the gaze cues and the arrow cues in RTs during the cueing task. A recent meta-analysis reported that gaze cues and arrow cues have comparable effects on attention orienting (Chacón-Candia et al., 2023). In adult studies, no difference between gaze and arrow cues in the cueing effects on attention orienting has been reported (e.g., Ishikawa et al., 2021). Moreover, a study involving participants aged 4–17 years demonstrated that gaze and arrow cues had equivalent effects on attention orienting as early as age 4 years (Aranda-Martin et al., 2022). In this study, children aged 7–10 years also showed similar levels of attention orienting with gaze and arrow cues. In addition to attention orientation, recent research has also examined attention disengagement. Wang et al. (2024) reported that social attention directed by gaze cues results in faster disengagement from the target compared to non-social attention directed by arrow cues. Although this study did not utilise eye tracking, leaving the target fixation duration unknown, the faster disengagement in social attention implies a processing difference beyond mere attention. If faster disengagement occurs in social attention as reported by Wang et al. (2024), it suggests that deeper information processing not explained by overt attention is taking place. A limitation of this study is the inability to measure looking time at the target. Future research using eye tracking is necessary to elucidate the mechanisms underlying the enhancement of implicit memory by gaze cues.

In this study, we developed a novel task to measure incidental social learning and demonstrated its utility with children aged 7–10 years. The incidental learning using the effects of gaze cues can also be applied to examining the spontaneous social cognition of individuals with autism spectrum disorder (ASD). ASD individuals often demonstrate social cognition abilities equivalent to those of the typical population in experimental settings, yet they frequently encounter social difficulties in everyday situations (Senju, 2013). This disparity has been attributed to the fact that experimental settings involve goal-directed cognition facilitated by instructions, whereas everyday social interactions require spontaneous social cognition. Research on the use of gaze cues has also reported intact gaze-cueing effects in individuals with ASD (DeJong et al., 2008; Kuhn et al., 2010; Rutherford & Krysko, 2008; Uono et al., 2009). However, it has been shown that individuals with ASD do not use gaze cues to infer mental states. Rombough and Iarocci (2013) developed a task to measure the extent to which children could follow eye gaze rather than a distracting arrow to infer mental states such as desire and intention. They found that autistic children were less inclined to prioritize and select information from another’s gaze. This result suggests that while individuals with ASD do engage in attention orienting towards others’ gaze cues, their spontaneous social cognition, particularly regarding the inference of mental states, is impaired. In our study's implicit learning task using the gaze-cueing paradigm, individuals with ASD may exhibit RTs comparable to those of typically developing individuals in response to gaze cues. However, they might not experience the same memory enhancement effects from gaze cueing. This task can measure the extent to which individuals with ASD spontaneously process cognitive information about objects in the direction of another person's gaze. In the current study, memory performance for targets that were not gaze cued remained at chance level, thus future research may need to modify the task to emphasize not just the target’s position but also its identity.

In conclusion, this study highlights the pivotal role of gaze cues in enhancing memory through incidental learning during childhood. It also provides valuable insights into spontaneous social cognition in children, suggesting an important role of other’s gaze cues in learning the surrounding environment. The value of following another's gaze and looking at the same object is likely linked to the motivation for cognitive processing in situations involving joint attention.

## Supplementary Information

Below is the link to the electronic supplementary material.

Supplementary file1 (XLSX 20 KB)

Supplementary file2 (DOCX 14 KB)

## Data Availability

Data used in the analysis are available in the Online Supplementary Material.

## Code availability

Not applicable. In this study, GUI-based software was used.
