# Supplementary material for: Gaze cues facilitate incidental learning in children aged 7–10 years, but arrow cues do not
Source: Psychon Bull Rev. 2025 Feb 14;32(4):1712–21. doi: 10.3758/s13423-025-02657-x (PMC12325541; doi:10.3758/s13423-025-02657-x)
Supplement: Supplementary file 2 — Supplementary file2 (DOCX 14 KB) [file 13423_2025_2657_MOESM2_ESM.docx]

**Performance in the cueing task**

　The average accuracy rate was quite high for each group (younger group: 96.58%; older group: 97.67%). The children included in the analysis could perform the cueing task as instructed.

　A three-way ANOVA (stimuli x cueing validity x group) was carried out on the response times (RTs). The main effect of cueing validity was found to be significant (*F*(1, 58) =20.833, *p*<.001, *ηp2* = .264). The children showed shorter RTs in the valid condition compared to the invalid condition. Also, the main effect of group was significant, showing faster RTs in the older group compared to the younger group (*F*(1, 58) =13.053, *p*=.001, *ηp2* = .184). The main effect of stimuli was not significant (*F*(1, 58) =0.00, *p=*.803, *ηp2* = .001). There were no significant interactions (stimuli x group: *F*(1, 58) =.063, *p=*.803, *ηp2* = .001; cueing validity x group: *F*(1, 58) =1.12, *p=*.294, *ηp2* = .019; stimuli x cueing validity: *F*(1, 58) =.438, *p=*.511, *ηp2* = .007; stimuli x cueing validity x group: *F*(1, 58) =2.827, *p=*.098, *ηp2* = .046).

**Memory accuracy**

A three-way ANOVA (stimuli x cueing validity x group) was carried out on the number of hits. The main effect of stimuli (*F*(1, 58) =6.019, *p*=.017, *ηp2* = .094) and the main effect of cueing validity (*F*(1, 58) =21.228, *p*<.001, *ηp2* = .268) were significant. Also, the main effect of group was significant, showing more hits in the older group compared to the younger group (*F*(1, 58) =8.108, *p*=.006, *ηp2* = .123).

A significant interaction effect between stimuli and cueing validity was found (*F*(1, 58) =26.438, *p*<.001, *ηp2* = .313). Post-hoc t-tests adjusted by Bonferroni’s method showed that there were more hits in the gaze valid condition than in the gaze invalid condition (*p*<.001). Additionally, in the valid condition, the gaze condition had more hits than the arrow condition (*p*<.001). There were no other significant interactions (stimuli x group: *F*(1, 58) =1.198, *p=*.278, *ηp2* = .020; cueing validity x group: *F*(1, 58) =.704, *p=*.578, *ηp2* = .005; stimuli x cueing validity x group: *F*(1, 58) =2.287, *p=*.136, *ηp2* = .038).
